# Daily and Cultural Issues of Postnatal Depression in African Women Immigrants in South East London: Tips for Health Professionals

**DOI:** 10.1155/2012/181640

**Published:** 2012-09-27

**Authors:** Titilayo Babatunde, Carlos Julio Moreno-Leguizamon

**Affiliations:** ^1^Health Visiting, Central North West London Camden Provider Services, London NW6 4DX, UK; ^2^Research in Health and Social Care, University of Greenwich, London SE9 2UG, UK

## Abstract

Postnatal depression has profound effects on the quality of life, social functioning, and economic productivity of women and families. This paper presents the findings of an earlier exploration of the perception of postnatal depression in African women immigrants in South East London. The aims of this research were twofold: firstly, to establish cultural elements related to postnatal depression through women's narratives regarding their daily life situations, including the nuances and complexities present in postnatal depression, and secondly, to help health professionals understand and acknowledge postnatal depression signs in these immigrant women and some of the cultural ambiguities surrounding them. The study used a qualitative approach mainly through the implementation of two focus groups. Thematic analysis of the women's narratives suggested that almost half of the participants in the study struggle with some signs of postnatal depression. The women did not perceive the signs as related to illness but as something else in their daily lives, that is, the notion “that you have to get on with it.” The study also highlights the fact that the signs were not identified by health visitors, despite prolonged contact with the women, due to the lack of acknowledgement of women's silence regarding their emotional struggle, household and family politics, and intercultural communication in health services.

## 1. Introduction

Depression is a major public health problem that is supposedly twice as common in women during the childbearing age as it is in men. [1]. It accounts for the greatest burden of disease among all mental health problems, and it is expected to become the second most prevalent of all general health problems globally by 2020 [2, 3]. According to [4] describe depression not just as a syndrome but also as an affective state that might be experienced by anyone at some point in their daily lives. According to them, depression is a mood disorder where mood refers to the prolonged emotions that colour psychic life. Affects, on the other hand, are the feeling tones or emotional states and their manifestations at a given moment. Mood disorders affect anybody without distinction of class, ethnicity, gender, education, age, or religion. 

Postnatal depression, a type of depression and, therefore, a mood disorder, is experienced by 1 in 10 women in the United Kingdom (UK) [5]. Technically defined, postnatal depression is constructed as an affective mood disorder often occurring in women up to one year after childbirth [6]. Furthermore, it is often characterised by feelings of loss and sadness and, sometimes, the loss of self-esteem [7]. The depressive scale of this disorder and its presentation ranges from mild depression requiring minimal intervention to puerperal psychosis which often requires multitherapy intervention, hospitalization, and long-term support [8]. 

Postnatal depression has profound effects on the quality of life, social functioning, and economic productivity of women and families [9]. The health consequences could also lead to adverse effects on the long-term emotional and physical development of the infant [10–12]. It is also predictive of child cognitive and behavioural disturbances at the age of 3 years [13, 14]. Moreover, failure by health professionals to identify postnatal depressed women often leads to safeguarding concerns for both mothers and infants [15, 16].

In general, health professionals and, in particular, health visitors in the UK play a vital role in identifying and supporting women who experience postnatal depression in the community. Their role includes supporting families during the period from the birth of the child to the age of five, thus enabling them to provide a prolonged period of contact and support for women affected by it. However, evidence suggests that most vulnerable women, including Black Minority Ethnic (BME) groups in the UK, do not always access or demand this service. This is because the symptoms are either overlooked or endured in silence by the women themselves in some cases, or they are not picked up by the health professionals in others [1, 5, 17–20]. In a study by [21] and in the study reported here, some women were reluctant to expose frailty and “stigma,” thus making it difficult for health professionals to provide adequate diagnoses or treatment.

There is a national recognition that over 6 million of the population of the United Kingdom experience some form of mental illness (depression); however, about 2 million of these do not have access to psychological therapies [22]. The fact that the BME population is growing in the UK and in South East London generates the need to explore and understand African women immigrants' perception of postnatal depression in order to improve service development and outcomes for them. For example, the African population is the second-largest ethnic group in Greenwich [23]. Women who are affected by postnatal depression often find themselves isolated and unable to return to employment. This problem is further exacerbated by the overwhelming evidence of the link between depression and domestic violence [24], and the differing conceptualisations of postnatal depression among health professionals [25]. This study presents the findings of an earlier exploration of the perception of postnatal depression in African women immigrants in South East London [26]. The aims of this research were twofold: firstly, to establish cultural elements related to postnatal depression through women's narratives regarding their daily life situations including the nuances and complexities present in postnatal depression, and secondly, to help health professionals understand and acknowledge postnatal depression signs in these immigrant women and some of the cultural ambiguities surrounding them.

## 2. The Multicultural Literature Concerning Postnatal Depression 

 The literature on mental health illness addresses the fact that people from diverse cultural backgrounds might display different constructions of mental health illness and, therefore, various ways of handling and coping with it. For example, some studies on depression among South Asian women, in particular Punjabis, have identified a cultural idiom called “sinking heart” which they experience as a result of excessive heat, exhaustion, worry, or a feeling of social failure [27]. Similarly, among women of African descent in America who had experienced postpartum depression (PPD) in the past, a study reports that they described and managed their depression in culturally specific ways, such as relying on their religious beliefs and the counsel of family members as well as keeping the depression a secret in the family [21]. The women also believed that only white women experience postnatal depression, as postnatal depression is considered a sign of weakness that does not represent a legitimate illness [21]. 

Some authors argued that the individual is bound by the rules of their culture which in turn shape and influence their behaviour [28, 29]. Similarly, cultural aspects of one's social system have a major impact on one's emotional life [28]. Major cultural differences influencing depression are family structure and dynamics, social organization, socially-sanctioned defence mechanisms, rituals, and social stresses [30]. Other cultural factors that may be important for a general understanding of depression include a distinctive language related to depression, the transmission of information among people about depression, and beliefs about healthcare and the healthcare system.

Prevalence rates of postnatal depression vary widely from culture to culture. Studies in developed countries report prevalence rates of 10% or more for postpartum depression [31]. In developing societies the figures are variable. Postnatal depression is thought to occur three times more in the developing societies than in developed ones [32], for example, in Khayelitsha, Cape Town, in South Africa, the prevalence rate of major depression was reported to be 34.7% at two months postpartum [32]. Other African studies that have looked at postpartum women have dealt with the prevalence of psychological distress in general rather than focusing on major depression or postnatal depression [33–35], which looked at prevalence of major depression at six weeks postpartum in Uganda, found a figure of 6.1%.

Similarly, the literature on mental health illness addresses the fact that aspects such as perceptions and attitudes towards depression in different cultures may affect help-seeking behaviour and access to treatment [10, 11]. Most studies in the literature regarding women's health-seeking behaviour coincide, for example, in pointing out that BME women tend to rely on family and religion as their main coping strategies. In the same way, for some women, postnatal depression is not perceived as an illness, yet they recognise the need to seek spiritual intervention. Equally, additional findings suggest that some black Caribbean women face difficulties describing or talking about perinatal depression due to their tendency to underreport their psychological feelings. Thus, barriers to health-seeking behaviour relate very much to the reluctance of some BME women to discuss problems as well as the way in which problems are dealt with [21, 27, 36, 37]. 

Another significant and controversial aspect of the literature, particularly in regard to the UK, relates to the diagnosing of postnatal depression among BME women through the Edinburgh Postnatal Depression Scale (EPDS). The EPDS is a psychometric measuring tool [38]. It comprises a ten-item self-rating questionnaire which is administered by health visitors approximately at eight weeks and at twelve months after childbirth. The EPDS is used by health visitors in the community. Following its validation for use in the UK it was implemented across the country by health visitors as a universal method of identifying mothers who were at risk of postnatal depression [39] argued that EPDS was originally designed as a screening test and was not intended as a diagnostic tool. However, many GP practices have continued to utilise the questionnaire as a single psychometric diagnostic tool. Thus, the controversy in the literature regarding the use of EDPS displays two positions: the one that considers this tool as culturally insensitive for BME women [40, 41] and the one that argues that it is effective [42, 43].

The literature that considers the EPDS to be less culturally sensitive to the needs of women from black and ethnic minority backgrounds states that it does not translate into other languages, let alone cultures [44, 45]. These authors also cautioned against direct translations of the tool, pointing out that some cultures do not have a word for depression, and suggesting that other screening methods should be considered depending on ethnicity. It is argued that the use of standardised Western methods and diagnostic classification systems, even by local-but-Westernised investigators, may be culturally insensitive and could increase the risk of practitioners missing symptoms or signs prevalent in non-Western cultures [46, 47]. Using EPDS as the assessment tool for these women might result in them often being inappropriately diagnosed or misdiagnosed, leading to omission. Additional arguments related to this position in the literature claim that most research has been conducted in the Western developed countries [31, 48] and has not taken into account the range of different psychosocial experiences likely to be involved in childbirth, for example differences in rates of lone motherhood, the nature of marriage, family kinship, and variations in the support new mothers receive in different countries and cultures.

For those who consider EPDS an effective tool for diagnosing postnatal depression the main evidence comes via some empirical studies that have screened women to check prevalence and associated factors in two groups: Nigerian and Black Caribbean women reporting a significant level of diagnosis [42, 43].

On the part of the health professionals, the literature addresses various factors that could contribute to the lack of awareness, late diagnoses, undetected cases or, worse, excessive medicalisation of symptoms. For example, [28] in a study investigating the influences of cultural factors in relation to postpartum depression, found that mothers from different cultural backgrounds may display culturally explicit behaviours and actions when suffering from depression. Another author [49] argued that the way a person perceives and understands their health is related to the subjective cultural experience in her or his society. [50] posits the idea that all cultures are unstable and subject to daily variations, innovations and change. Similarly, [51] clearly demonstrates this in a study on how women understood and responded to depression according to their cultural understanding of the disorder. According to [30], culture can be understood as shared beliefs, learned values, and attitudes which shape and influence perception and behaviour. In other words, African women immigrants in the UK could be seen as a group of people who share history, religion, language, thoughts and, overall, the experience of being immigrants. Thus, how the cultural background of women is understood and constructed by the providers of health services and how these providers and the women communicate is a matter of great interest for researchers focusing on intercultural communication in the context of health services in various multicultural societies.

Ultimately, although postnatal depression affects all women regardless of ethnicity or social class, additional contributory risk factors include social exclusion, deprivation, and relationship complexities [52]. Thus, despite all the attempts in the literature to explain the causes of this illness, no single factor has been successfully identified as its cause. On the contrary, as discussed above, several explanations have been put forward by the literature. This qualitative study presented here [26] expects to add to this ample range of explanations in the literature on postnatal depression particularly among African Women Immigrants in South East London.

## 3. Materials and Method

The qualitative study reported here used focus groups as a means of collecting data from participants. The focus group is an in-depth, open-ended group discussion that explores a specific set of issues on a predefined and limited topic [53]. Focus groups within feminist work have been devised to elicit and validate collective testimonies and group resistance narratives. These testimonies and narratives have been used by women and could be used by any subjugated group “to unveil specific and little-researched aspects of women's daily lives, their feelings, attitudes, hopes, and dreams” [54, page 835]. They can also facilitate the identification of cultural values and are said to be valuable when researching ethnic minority groups [55].

### 3.1. Recruitment, Focus Groups, and Ethics

The group targeted for the study was all African women immigrants registered on the general lists of the health visitors of four health centres in South East London. Participants were contacted personally, through leaflets, and a phone call by the researchers but the gate keepers for the research were the health visitors. Twenty-six immigrant women of African background between sixteen and forty-five years of age were purposively identified and selected. They were identified by both the health visitors and the researchers. However after the selection of twenty six women, only seventeen were able to confirm and attend the focus groups (see Table 1 below). These were divided into two groups as seventeen people would had been a too large number for only one focus group. The women were then given the choice of the focus group they preferred to attend. There were nine in the first focus group and eight in the second one. The numbers of women for each group were within the standard methodological recommendation for a number of people in a focus group which can be from six to nine people in accordance with [56]. Furthermore, the purposive selection followed [57] suggestion that a deliberate nonrandom sampling should include a group of people with particular characteristics, in this case: immigrant women of African background.

During the two focus groups a psychologist was in attendance and acted as a research cofacilitator, taking field notes. Two nursery nurses were also in attendance. They help to organise play activities for the toddlers who came with their mothers and with the signing of the consent forms. Further, each focus group lasted for about two hours with fifteen-minute breaks in between. The two groups were held in a Children's centre, a familiar environment for the participants. Having a cofacilitator meant that the two other researchers were able to lead the discussion while the cofacilitator was free to take notes and assist with the subsequent transcription of the data collected [53]. All activities took place in the same large room and no major disruptions took place.

The main inclusion criteria which applied to the women participating in the research were (i) women in the postnatal period who had delivered a baby up to a year earlier, (ii) immigrant women who identified themselves as being of African descent between the ages of 16 and 45 years (the age range within which women's fertility and reproductive capacity is at its peak), (iii) women who spoke and understood English, (iv) women whose bab(ies) were in a good health, and (v) women who lived in the South East (women with little children are always busy and will hesitate to attend any group if the distance is more than two miles) and (vi). The inclusion criteria were kept flexible. However, strict exclusion criteria were: (i) women who had children subject to the child protection procedures, (ii) women aged over 45 years, and (iii) women who were under 16 years of age (parental consent issues). 

Ethical approval for the research was obtained from the South London Research and Ethics Committee. An application was made through the Integrated Research Application System (IRAS). The National Health Service Trust also gave the required Research and Development (R&D) approval. Ethical Research Committees in the UK endorse the Declaration of Helsinki which seeks to minimize harm towards research participants [56]. Thus, in this case voluntary informed consent, confidentiality and anonymity were explained to the women at the beginning of each focus group. They also were informed about their right to withdraw at any point should any discomfort arise [56]. Participants were picked up from their homes and taken back. They were also given a gift voucher as a token of appreciation for attending the focus groups.

### 3.2. Data Collection and Data Analysis

In order to collect women's narratives, the logic of qualitative inquiry was used. In the discussion on research into sensitive issues such as postnatal depression, and the differences between qualitative and quantitative studies, an important phenomenon to recall is the *ergodic* hypothesis as postulated by George Devereux in the seventies [58]:

*“The analysis of a great number of relatively superficial facts—ampleness—provides exactly the same insights as the in-depth analysis of only one phenomenon. Ampleness is depth, rotating 90 degrees in horizontal position; the depth is ampleness if the 90-degree turn is on a vertical position. The equivalence of the two phenomena takes exact root in the Ergodica hypothesis… In fact, under this premise, it is postulated that the same results are obtained whether throwing X number of coins simultaneously (for example, 10 coins at the same time) or only one coin X number of times (1 coin 10 times). In the context of social research, this yields an equivalence for a survey with one thousand answers of yes or no type questions, and three in-depth interviews of, say, 4 hours” (page 108).*

Following the qualitative approach, the researcher asked the women at the very beginning of the two focus groups about their marital status and the kind of support network they had at home. Studies have shown that women's marital status and the kind of support network they have are significant risk factors that may predispose a woman to postnatal depression [52]. Similarly, their educational background or employment status may also affect their perception and the way they describe postnatal depression. Thus, a basic task here was to map the sociodemographic characteristics of the participants as illustrated in Table 1.

Regarding group dynamics while the focus groups were implemented two main observations emerged. First, according to [59], in a group setting group norms may silence dissent; indeed, in one of the focus groups a social hierarchy was observed. One of the women tended to dominate the group, silencing any mention of family problems and thus intimidating the less confident women within the group. This was dealt with by inviting the less vocal among the women to contribute their experiences. An attempt was also made to contact these individuals soon after the group to ensure they actually had a voice in the data collection process. Secondly, an observation that became pretty obvious during the two focus groups was that those participants who were more educated, up to degree level, were more vocal and their perception of the symptoms of postnatal depression were freely expressed whereas those who had achieved GCSE level were less expressive. In fact the most vocal and educated women in the first focus group contributed to within—data saturation [60], since they articulated many themes setting the trend, that reemerged in the second focus group and, later, during the analysis of the data. 

Following each focus group the data were transcribed verbatim using a thematic analysis. As a qualitative research practice, theme analysis comprises the process of examining a piece of data as many times as possible until patterns or themes emerge. A theme, broadly speaking, is nothing more than a cluster of similar units of meaning that had been stated by the different participants in the focus group.

In the particular case of the study reported here, the thematic analysis was performed by reading the women's statements several times. Initial coding of the transcripts was performed with the goal of remaining open to all possible interpretations. Codes either stored information about patient demographics or were far more analytical, representing links between the data and an idea [61]. Codes were made as descriptive of the participants' experience or thoughts as possible. Thus, once the data were sorted by units of meaning, the themes were identified. The themes were grouped and examined in all cases to make sure that all the descriptions of each theme had been captured and compared.

Table 2 shows some of the most significant stages in the thematic analysis of the data (narratives). The first column on the left shows some significant excerpts from the statements provided by the women. The second column identifies units of meaning based upon the narratives. The last column shows the emerging themes from the data. However, this paper moves from just description of the themes to examine how the themes are interconnected according to Pope et al. [62].

## 4. Results

The results discussed here present the data through the main themes and literal quotations (narratives) as stated by the women regarding events, episodes, points of view, settings, and comments related to the main focus of this research: postnatal depression among a group of African women Immigrants in South East London. The main themes accordingly were: responses to their pregnancy, feelings before and after giving birth, social support or the lack of it, feeling alone, lack of information about health services, poverty, signs of postnatal depression, and not coping with their situation.

### 4.1. Responses to Pregnancy

Participants were asked to reflect on when they first realized they were pregnant. This was to allow them to consider what their reaction had been at the time and to describe their emotional feelings. Ranging from the ones who experienced difficulties in seeing the good side of being pregnant to the ones who were happily surprised, the main trend in this theme was that being pregnant is always a good thing for all women but particularly for African women immigrants who are married or cohabiting. It was considered that If a woman is not married, nobody cares whether she is pregnant or not; once married, the expectation is that she is ready for the responsibility and that includes having babies. For example, describing her experience, a first-time mother educated up to secondary school level said:

*“When I first became pregnant, I was scared, during the pregnancy it was quite difficult for me because I felt ill, sick all the time, lost a lot of weight and this happened throughout the pregnancy. It was really hard.”*

Participants also reported their experiences and feelings during the antenatal period that made it extremely difficult for them to see the good side of being pregnant. As another participant clearly stated:

* “So I realise it was the pregnancy, it was mixed feelings… I'm very happy… I'm very healthy but not so happy as there are all other stuff going on in my mind, such as not being with the father or married to him, only empty promises, happy but sad.”*

### 4.2. Emotional Feelings before the Birth of the Baby

The women reported emotional feelings associated with being pregnant and almost all participants reported these. For example, a woman reported the emotional stress she incurred by getting pregnant while still at school and not finishing her secondary school education. The participants described feelings of being sad, angry, annoyed, and irritable, not with anyone else but with themselves; they blamed themselves for being pregnant or evaluated the tasks before them and assessed their ability to cope with the tasks. As illustrated by another participant:

*“This was my first baby, I was afraid and also I don't have family here… and was crying all the time and very lonely.”*

### 4.3. Emotional Feelings after the Birth of the Baby

The cultural ideal for describing the postnatal period was strongly endorsed by and pervasive amongst participants. This was accompanied by tacit acknowledgement that the actual experience of many women would fall short, leading to disappointment at unfulfilled expectations and potential risk of postnatal depression. Rest was considered a necessity following the demands of pregnancy and childbirth but the participants were totally disappointed as the shock of having a crying baby hit them. They received no help from husbands or family members and had to cope with the demands that came with this new, vulnerable child, who needed help and care constantly.

*“So I suppose I was happy to become a mum, emotionally I was sad, and I……… but I just kept crying and she was quite shocked and she spoke to me and said to me “Oh it's OK to cry because if you don't cry you can become very depressed. And I said Why am I crying?” I couldn't think why I was.”*

These feelings were attributed to practical problems: for example, having little opportunity to rest, and lack of support at home. These are serious issues in women's lives so, for them, it was a case of finding a way to cope with feelings nobody else understood or which they could not explain to others for fear of being labelled or misunderstood. As various participants stated:

*“so the first week was very difficult for me to cope with changing him, feeding him, it wasn't easy, it was my first time, so I would cry sometimes; and not until when my mum came, things were a little bit easier for me. But I couldn't really cope with the emotions. Sometimes like I said, it was mixed feelings.”*

Here symptoms are very much associated with a state of unhappiness following delivery, although by no means all recognised it as an illness—postnatal depression—or a requirement for treatment by healthcare professionals.

### 4.4. Social Support or the Lack of It

The participants, being relatively recent immigrants to the UK, spoke of the difficulties they faced in this country, which promotes equality between men and women. On average these women have been in the UK for about five years. However, the reality in their homes is different. As narrated by various women, they did all the household chores regardless of the number of days that had elapsed since the birth. They received no help from their husbands nor even from their mothers-in-law; in fact, some women reported that the in-laws made matters worse. As one of the participants strongly stated:

* “…… I mean having a baby is difficult but I did all the cooking, my in-laws were around, I was frustrated, nobody helped me do anything for six months, I changed the baby's diapers all the time, nobody helped me once. I was cooking, I was going to the market, and there was no help for me. I cried a lot, felt rejected by my husband, So by the time I got pregnant the second time, I wasn't looking forward to having the baby, I was……….”*

* “[The in laws] would come around just to see the baby, ask me how I'm doing, and leave when they should actually be helping me, but they didn't do any of that. So that was one of the things that really got to me, like I've got help here but….”*

And although most of the participants had husbands they emphasised that they did not help at all. They described it as an “African thing” and called for awareness training for some African men in order to educate them on issues of the postnatal period.

* “I mean for African perspective, this is how we do it. We do it this way. Why do you want to do it that way? So then whatever you want to say has been short out of you or stays in you. If you want to with mum, I'll deal with the person who's trying to tell you what you have to do.”*

### 4.5. Being Alone with Feelings

For some participants, their postnatal status meant that they were unable to escape and thus had to stay and endure the hardship. Participants indicated that a major source of distress was being unable to share their emotions with their immediate family for fear of being seen as a failure. Some participants spoke about talking to their husbands about their emotions while others did not hold any hope that it would improve the situation as the men do not see their role as providing help even though they live in the UK. Participants felt there was no generally accepted person to talk to; one participant pointed out that, even when the health visitors come, the mother-in-law is sometimes present, thus making her (the participant) uncomfortable about displaying her emotions in front of the mother-in-law. The quotations below are illustrative of this theme:

*“It's something nobody else can help you with. Like someone can help you with the baby and help you with other things, but the way that you're feeling, you don't get help for that.”*

*“That I'm going mad, mentally and sometimes I'd be crying… the baby and I will be crying, and sometimes I feel like throwing the baby out, but I can't.”*

*“I don't know. Maybe it's my culture, I don't know. It could be cultural. I can't imagine myself going to my mum, or my mother-in-law… probably I can say to my mum, but I still didn't, I just couldn't imagine saying to somebody, “Oh do you know what? I'm really struggling, I'm really down…” It just sounds odd. It's just not… it's not something that you do, you just… Everybody expects you to get on with it and you get on with it.”*

### 4.6. Lack of Information

Participants pointed out that there is a general lack of awareness among UK health visitors and healthcare professionals in identifying when African women immigrants are in distress or are crying out for help. A woman remarked that she was given a questionnaire (referring to the EPDS), which to her made no sense:

*“but they didn't really give me some of the other information that as a new mother I would have found really useful, without me having to look on the Internet or buy a book.” “To me, the information given by the health visitor did not help me at all; I had to search the Internet myself for answers to some of my questions.”*

The same woman pointed out threats to her mental health arising from her postnatal experience. Her inherent vulnerability and the potential consequences of her distressed mental state were not adequately dealt with, probably because of the professionals' failure to capture the struggle and nuances, in this case, of what this woman was going through. This participant also felt she needed more information from the care providers that would have helped her cope better after the birth of her baby.

### 4.7. Poverty

Another theme repeatedly raised by the participants is the issue of social class and working-class women. Money or poverty seemed to be a major concern for participants. There is the issue of the father/partner, mother or both working long hours and not earning enough. There were also issues about providing for the family, because social welfare provision is not a practice in their country of origin or they do not have recourse to public funds due to their status as recent immigrants. As a result both parents have to provide for the family and that is the acceptable norm. This is harder in the United Kingdom because of childcare arrangements. As another participant stated:

*“With this baby I felt really depressed because I didn't want to have another child because I raised my other daughter on my own. She's nine now so I thought that's the hard work… I didn't want to do it all over again, so all the time I was up and down, some days I feel ok some days I feel really depressed.”*

### 4.8. Signs of Postnatal Depression

Participants described some of the signs of postnatal depression; however, the main theme here is that they would not dare admit to their families or relatives that that was how they were feeling at that moment in their lives. The fears of being seen as a failure or being stigmatised or labelled as mad were some of the reasons women kept their feelings to themselves. As one participant added:

*“It was like a torture. I mean I was screaming at the midwife so I was just screaming, I cried, postnatal depression, so I said No, I want to go home.”*

### 4.9. Not Coping

Participants also stated that there are instances when they feel out of control—not coping—and even sometimes the feeling of “losing it” completely. Furthermore, almost all of the seventeen participants agreed that there is a problem with the present system of provision of healthcare services for African women immigrants in capturing these emotional distresses. They also recognised that they do not speak to professionals about their true emotional feelings for fear of being labelled depressed, which is a taboo or a stigma in both their country of origin and to a lesser extent in the UK. As another participant stated:

*“so the urge to want to speak up and say Hey, I actually need help… you know, I'm not coping here, superwoman, you know, I can do this, but you're not. Inside you're not.”*

## 5. Discussion 

The main findings were related to: response to participants' pregnancies, feelings before and after giving birth, social support or the lack of it, feeling alone, lack of information about health services, poverty, signs of postnatal depression, and not coping with their situation. And as mentioned before, these were women's statements that reached a good level of within-data saturation [60], since they emerged rapidly from the first focus group and kept being repeated in the subsequent focus group. In order to synthetize them, the statements are discussed in terms of the direct experience of the women (feelings before and after giving birth, signs of postnatal depression, a feeling that they are not coping with the situation, feeling alone, and expectations of and responses to their pregnancy) and, also in terms of perceived support from families (husband and mother-in-law), community and health services (lack of information and health visitors). 

The majority of the statements articulated by some of participants and related to their direct experiences indicated symptoms associated with a state of unhappiness following delivery, although by no means all recognised it as an illness—postnatal depression—or felt they required treatment by healthcare professionals as addressed too by the literature [10, 11, 21, 27, 36, 37]. As one of the participants stated powerfully, rendering it invisible, it is “something that you have to get on with.” However, their narratives showed features such as distressed states manifested by sadness, irritability, anger and unhappiness, all of which characterise the medical construct of postnatal depression. So, certainly, the narrative of those states was comparable to the criteria for a diagnosis of postnatal depressive disorder but, as seen, these become invisible because they are not identified as postnatal depression as such. 

Further descriptions of the women's experiences related to their emotional feelings when facing lack of social support. They perceived themselves as drained, which made them feel emotionally down. Additional units of meaning here included “caring for my baby alone”, “cooking for the family,” “worrying about my finances” and “looking after the house.” So, as the literature also shows, the lack of all types of social support cannot be ignored here as a major risk factor for postnatal depression [31] particular in immigrant women, whose close relatives are absent.

In their narratives women expressed emotional distress as something different from physical illness, and that one may cause or influence the other. Their descriptions fitted in many ways the psychosocial model of health as this conceptualises health as a bio-psychosocial issue [58, 63], rather than an exclusively biomedical issue. They acknowledged that emotional distress was a product of their social conditions combined with their own individual issues. 

According to the literature two major stressors for mothers of newborns going through the postnatal period are: (i) recovering from the immediate physiological changes caused by delivery; (ii) returning to functional status (which rarely returns to normal prior to six weeks) during the days and weeks after delivery [64]. Thus, discussions with new mothers during this period must include those two stressors. In the case of women in the two focus groups, most of the stressors expressed by the women are included among the themes as, for example, mental stress such as loneliness, unmet expectations, birth plan disappointment and abandonment, and external stressors such as crying baby, sibling care, lack of support, and financial concerns. Of course, while women in the study reported here did not experience all the stressors at once, most experienced some simultaneously. Experiencing multiple stressors during the postnatal period can lead to sleeplessness, fatigue, and irritability, which were also part of the narratives. Studies have shown that these multiple stressors are risk factors for postnatal depression [31, 65].

From the women's narratives it was apparent that their African cultural background has a bearing on their help-seeking behaviour. Although some of them felt sad, unhappy, and stressed, they kept their feelings to themselves because, culturally, to admit having problems coping with the after-effects of childbirth is probably a sign of failure or weakness in front of the extended family—husbands, mothers-in-law, and others. Thus, as [66] argues, the culturally appropriate terminology for depression seems to be an issue for further research here. 

Also, as expressed in their narratives, the fact that women may choose to make their emotional problems invisible to health professionals could find explanation in their cultural backgrounds and their status as newly arrived immigrants. As the literature addresses, among various factors, cultural background is one that could lead to some women being undiagnosed [18], particularly African immigrant women in the UK. Women from black ethnic minority backgrounds may not be diagnosed with postnatal depression because of their fear of being stigmatised and their cultural perception and understanding of postnatal depression [5, 17].

The cultural background of immigrants as a factor in the concealment of postnatal symptoms is important because it is vital to recall that cultural practices are not frozen activities that determine unequivocally the behaviour of an individual. Culture is reenacted by individuals daily and is responsible for the embedded ambiguity in the way they react. Immigrants are neither in the old familiar place nor fully tailored to the new place—including, among other things, their access to or demand for health services that are mainly biomedically oriented [28, 29, 67]. So the common conceptualization of culture by health services as a frozen element that determines people's behaviour, attitudes, or understanding of the lifeworld should be modified to that of daily enactment and ambiguity which needs to be understood by the health professionals in each specific case [67]. Here, there is a need for skilful health professionals to work with women who experience this cultural ambiguity regarding postnatal depression. 

As some of the participants narrated, counting on their own mothers for support seems to have helped some participants cope with the distress they faced during this postnatal period although not in all cases, as a few never had the opportunity for their own mothers to be present due to their immigrant status. Similarly, as described in the literature [21], turning to prayer in order to cope with their stress levels was seen by other participants as a coping mechanism, particularly for those with strong religious beliefs.

Interestingly, the two focus groups welcomed the idea of a group such as the focus group which allowed them to communicate their internal emotional struggles and feelings with women who were going through the same experience.

Another important and recurrent finding as presented in the narratives of the participants in the two focus groups was the mention of the mother-in-law as one of the sources of unhappiness in the dynamics of the household once the baby has arrived. As described by participants in some African groups, once a woman gets married, it is expected that, in the next few months, she will become pregnant. The absence of babies causes unfulfilled expectations in both families—the woman is seen as an alien and the longer she remains without a child the more sadness she experiences. She receives unfriendly comments from her mother-in-law and other members of her husband's family, and not even the husband will intervene in this matter. In the study reported here, this fact was revalidated by the comments of two of the participants who were married for about a year before conceiving. So, when the pregnancy finally came they were elated. This happiness, however, was cut short by the arrival of their mothers-in-law, who did not help them at all but rather caused them distress.

After the mother-in-law, the second most culpable figure seen as failing to provide any social support was the husband. And, although most of the participants had husbands, they emphasised that they did not help at all. They described it as an “African thing” and called for awareness training for some African men in order to educate them on issues of the postnatal period. 

Ultimately, without denying the biomedical mechanisms present in postnatal depression, the interesting issue to discuss here in closing this discussion is the fact that the women through their narratives volunteer the sources of solutions based on the psychosocial model of health [58]. Social support from family, practical and emotional support from partners and having someone to talk to were unanimously expressed as the “remedies” for what they were experiencing: isolation and self-inflicted suffering endured in silence because of the fear of being labelled and stigmatised as emotionally unstable by the immediate family (partners and in-laws). This overwhelming result found by the study reported here was also very well addressed by the literature on postnatal depression [21, 27, 33–37].

### 5.1. Research Implications for Clinicians and Health Providers

Contextualising the most important findings discussed above (acknowledgement of postnatal depression, social support, and emotional distress, cultural identification and coping strategies) in some of the recent discussions in social sciences, three issues are addressed below, particularly for health professionals to consider when offering health services for people of different cultural backgrounds, in this case African immigrant women. The dialogue between social sciences and nursing science is imperative in the context of framing health as a bio-psychosocial, cultural and political issue rather than a biomedical one. 

The first issue is that of the acknowledgement of suffering, which has been addressed by medical anthropology. In particular, [68] have addressed the issue of the role of language in the incommunicability of pain and suffering. As the authors brilliantly state:

*“To be in pain is to be certain about this knowledge. To be asked to react to another person's pain is to be in doubt about its existence. From the perspective of theories of social suffering, such a preoccupation with individual certainty and doubt simply seems a less interesting, less important question to ask than that of how such suffering is produced in societies and how acknowledgement of pain, as a cultural process, is given or withheld. After all, to be ignorant or incapable of imagining another person's pain does signal blindness in moral sensibility in the same way in which the incapacity to acknowledge that pain does” (page.xiii).*

Unquestionably, failure to acknowledge that African immigrant women to the UK are struggling in an ambiguous way with their emotions in their postnatal period calls into question the understanding of suffering and pain by the health services and health managers trapped in frameworks of accountability and quantitative indicators. Can we imagine the health services, managers and professionals acknowledging the statement “it is something that you have to get on with” with something like: “No, it is not something that you have to get on with!”? Certainly the health services could show these women that their struggle has alternative solutions.

The second issue has to do with the contemporary conceptualisation of power. According to this notion, power is a multiple force cross-cutting human relations and therefore spaces such as the domestic arena and even the clinical space. Power, as described by the narratives of the women, plays a role in the dynamics of their households through the family politics and certainly underpins most of the features the themes are pointing out such as domineering husbands and lack of support for the women, powerful role of the mother-in-law, isolation, and self-inflicted suffering by the women themselves who remain silent about their stress. 

So, an understanding of the family politics—that is, how all the members of the extended family display and wield power around the pregnancy issues as described by these African women immigrants—is something that needs to be incorporated in the bio-psychosocial model of health services when working with them. These compelling features should make it clear to the health professionals that they are not working with the assumed normality that is the reconstructed British nuclear family. Of course, this is not say that such politics are not also played out among the reconstructed British nuclear family. It is just that the politics differ between different groups. 

The third issue to be discussed here has to do with intercultural communication in the health setting between these immigrant women and health professionals. As demonstrated by the narratives, on the one hand the women's concealment of the emotional stressors from themselves, complemented by the fear of failure, generates some self-inflicted suffering. On the other hand, the family members' disregard and stigmatisation of the stressors, along with the professionals' failure to acknowledge the cultural ambiguities, adds to the women's suffering. The fact that professionals often report women as being well because they have hidden their internal turmoil is significant evidence of the lack of awareness of intercultural communications among health professionals in observing culture and power at play. And this could be the result of the lack of education among health professionals in the discussion of contemporary social sciences as well as the new discussion on compassionate care.

Combining the features described above along with more specific tips for health professionals, it is evident that the main one comes via the approach used by health service managers and professionals, at least in the in the UK, to conceptualising health. If health is a bio-psychosocial issue [63], the acknowledgement of the suffering of these immigrant women, the household politics they face and the intercultural communication between them and the health professionals are issues that can definitely be accommodated. But this ethical and academic decision lies in the hands of the health services. 

Feminist research has demonstrated that the best outcomes in improving women's lives come from the work done with both men and women and not just women [69]. Health professionals probably need to understand that postnatal depression in African immigrant women is not the exclusive issue for these women, as the research study reported here has tried to demonstrate. This has implications for health professionals working with postnatal women since their experiences should extend to and include the understanding of the entire family and not just the postnatal women. Again, as [70] points out when outlining the social suffering theory, suffering and pain as in the case presented here of postnatal depression, inasmuch as they are health and social problems, needs to take into account not only the individual but also his or her networks.

## 6. Limitations of This Study 

There were four limitations in this study which eventually could relate to the literature concerning qualitative research with BME groups or other vulnerable groups [71–74]. First, when carrying out research on health issues, the researcher has to abide by the ethnic self-identification of the person that the research is about rather than engage in labelling. In this case the women who participated in the study reported here self-identified themselves as African immigrants to the UK in the last decade. 

Second, regarding the issue that research with people of different cultural backgrounds should ideally look for same cultural matching between researchers and participants has been challenged. In particular, [73] argues that more than ethnic matching, researchers and research participants should look for a mutual or shared understanding regardless of their cultural background. In the case of this research there was an effort to combine both shared understanding and ethnic matching. Thus while the ethnicity of the three main researchers involved in the data collection were, respectively, African, English, and South American, the gender mix involved two females and one male. So any possible limitations in the research reported here could be related to the these cultural backgrounds.

Third, the literature on qualitative research becomes controversial where data saturation regarding focus groups is reached. While some authors mention that this is obtained between three and six focus groups, [74] other authors [75] view it as a reemerge of a theme or a topic even if it is in one focus group. In the particular case of this research, data saturation took place from the first focus group due to the influence of the most vocal and educated women. However, as was also indicated, this trend was countered by taking into account the statements of the less vocal women. 

Fourth, trustworthiness and rigour in qualitative research as suggested by [72] should come through the dialogue or agreement between, on one hand, the reader of the research, and on the other, the detailed description of how the research process was conducted by the researcher. In the case of this research the materials and methods have been described as carefully as possible so that readers can follow the process by which this research with African immigrant women was conducted. Equally transferability as part of trustworthiness and rigour in qualitative research comes through the description of the specific context in which the research has taken place [72]. Thus a possible limitation of this research could be that some of the situations discussed (acknowledgement of postnatal depression, social support and emotional distress, cultural identification, and coping strategies) in African immigrant women relate only to them while the same or other similar aspects discussed relate to other women from other cultural backgrounds. However this was beyond the scope of this research. 

## 7. Conclusion

The study, using the logic of qualitative inquiry, the *ergodic* hypothesis as postulated by Devereux [76], showed that African immigrant women in South East London received little practical and emotional support before, during and after delivery of their babies. As the narratives of the study illustrated, these women suffer and cope with their emotional distress alone and in silence, magnifying their suffering. They see emotional distress as something different from physical illness and very much framed in the bio-psychosocial model of health. Their narratives also allow us to infer that support from the immediate extended family (mainly in-laws and husband) is inadequate and these family members barely understand what the mothers are going through. The same lack of acknowledgement and understanding was observed by the healthcare services. 

As demonstrated by the study, on the one hand, the women's tendency to keep all the emotional stressors to themselves complemented by the fear of failing generates some self-inflicted suffering. On the other hand, when the family members ignore the stressors and stigmatise the mothers, and the professionals fail to pick up any of these cultural clues, the women's suffering is compounded. The fact that professionals often report women as being well because they have hidden their inner turmoil is significant evidence of the lack of acknowledgement of the suffering the women are going through as well as the lack of awareness of intercultural communication between health professionals and the women, in this particular case African immigrants in London. There is a need for health professionals to embed cultural ambiguities in their daily work routine, as culture is not a frozen equivocal determinant of peoples' lived world. Again, in this particular case involving immigrant women, can we imagine health services, managers and professionals acknowledging the women's statement “it is something that you have to get on with” with something like: “No, it is not something that you have to get on with!”? Their struggle has alternative solutions. The discussion of contemporary social sciences could help here immensely as well as the new discussion on compassionate care.

Simultaneous with the acknowledgement of the women's suffering, it was seen as important that the health professionals understand the family politics of any household. The inclusion of family politics in the bio-psychosocial model of health services is imperative. As the narratives showed, health professionals need to understand that, in a multicultural society, there is more than one assumed normality besides the reconstructed British nuclear family. Taking advantage of multiple readings into this will help the health services. But, as has was already been stated, this and other ethical and academic decisions lie in the hands of the health services.

## Figures and Tables

**Table 1:** Participants' socio-demographic characteristics.

Country	Number	Education	Marital status	Employment status
Nigeria	11	2 have bachelor degree, 9 educated to a general certificate for secondary education (GCSE)	9 married and 2 single parents	1 self-employed, 1 on maternity leave, 9 unemployed

Ghana	2	1 above advanced level	One married, the other single parent	1 looking for job
		1 studying for degree		1 student

Kenya	1	Studying for a national vocational qualification	Has a boyfriend but not living together	Student

Somalia	1	General certificate of secondary education (GCSE)	Husband abroad	Unemployed

Sierra Leone	2	General certificate of secondary education (GCSE)	2 single parents	2 unemployed

**Table 2:** Themes arising from data analysis.

Illustrative quotations from women	Unit of meaning	Emerging themes
*“When I first became pregnant, I was scared; during the pregnancy it was quite difficult for me because I was sick all the time. It was really hard*.*” *	Sick all through pregnancy; anxiety about job loss and discontinuation of education	Responses to pregnancy

*“This was my first baby, I was afraid and also I don't have family here… and was crying all the time and very lonely*.*”* *“So I realise it was the pregnancy, it was a mixed feelings I'm very happy, I'm very healthy but not so happy as there are all other stuff going on in my mind, such as not being with the father or married to him only empty promises, happy but sad.” *	Crying a lot; sadness; lonely; unexpected expectation	Emotional feelings before birth of baby

*“OK. For the first one, after I had the baby, I didn't have much help for the first one, and I felt really isolated, and even though I had people around, but they were not helping me, I was doing things for them, even though they should have been doing things for me because pregnancy is difficult… I mean having a baby is difficult but I did all the cooking, my in-laws were around, I was frustrated, nobody helped me do anything for six months, I changed the baby's diapers all the time, nobody helped me once. I was cooking, I was going to the market, there was no help for me. I cried a lot, felt rejected by my husband.”*	Very tired/baby cried all the time; mixed feelings; isolated; rejected by husband; depressed	Emotional feelings after the birth of the baby
*“OK. I'm Ghanaian, I will go straight to the question which is my emotional feelings. When I first realised I was pregnant, it was a mixture of excitement and sadness because of my own situation that I was going through. So I suppose I was happy to become a mum, emotionally I was sad, and I……… but I had sessions of uncontrolled cries and could not explain why.”*	In-laws causing rift	

*“When I found out I was pregnant, I was really happy because we'd been trying for some years, and I was actually in the process of finding out if there was something wrong, and then I went to the Chinese man for my hay-fever, so we're always like… we'll never know whether it was nature or if it was the herbs, and we were really happy*.*” *		
*“… pregnancy is difficult… I mean having a baby is difficult but I did all the cooking, my in-laws were around, I was frustrated, nobody helped me do anything for six months, I changed the baby's diapers all the time, nobody helped me once. I got pregnant the second time, I wasn't looking forward to having the baby, I was………”*	In-laws' interference when pregnancy not forthcoming, Husband left	Social support or the lack of it
*“… And those, all those things, all the culture and all the things that happen, make the afterbirth very difficult, something that a bright person, very vibrant and passionate, all of a sudden you're just like demoted…”*		

*“Well for me, being pregnant is always an exciting experience for a mum, you know, knowing that you're going to bring forth someone, a child, that can change the course of the world, so I was excited.”*	Source of joy; happy; long-awaited surprise	Expected pregnancy

*“It's something nobody else can help you with. Like someone can help you with the baby and help you with other things, but the way that you're feeling, you don't get help for that*.*” *		
*“That I'm going mad, mentally and sometimes I'd be crying… the baby and I will be crying, and sometimes I feel like throwing the baby out, but I can't.”*	To express feelings is a sign of weakness	Being alone with feelings
*“I don't know. Maybe it's my culture, I don't know. It could be cultural. I can't imagine myself going to my mum, or my mother-in-law… probably I can say to my mum, but I still didn't, I just couldn't imagine saying to somebody, “Oh do you know what? I'm really struggling, I'm really down…” It just sounds odd. It's just not… it's not something that you do, you just… Everybody expects you to get on with it and you get on with it.”*		

*“I felt that (health authority) spend a lot of money on teaching you to breastfeed in the hospital, but the people who were trying to teach me I don't think were very good and I felt like they were pressuring me a bit as well, but they didn't really give me some of the other information that as a new mother I would have found really useful, without me having to look on the internet or buy a book. And speaking to some people doesn't help because they just make you feel like it's just your baby crying…”*	Not enough information of what to expect as a first-time mother	Lack of information

*“I'm not that After my first baby I think my depression was caused by… because after the first child I wanted to get a job and to start working maybe.”* *“Money, yeah, but I'm trying my best, you know, to do all I can do, you know. type of person that I want to wait for my husband to put the money on the table all the time, you know, whatever I think I can do I do to get extra money…” *	Hardship experienced in terms of finance, shopping and working/extra income	Poverty

*“That I'm going mad, mentally and sometimes I'd be crying… the baby and I will be crying, and sometimes I feel like throwing the baby out, but I can't*.*”* *“It was like a torture. I mean I was screaming at the midwife so I was just screaming, I cried, postnatal depression, so I said No, I want to go home*.*” *	Failing to admit that they are depressed as it is taboo to admit to such	Signs of postnatal depression

*“Very tired/baby cried all the time*.*” *		
*“Mixed feelings—happy at times and sad sometimes*.*” *	Crying at times but only in secret as cannot be seen to be failing	Not coping
* “Feeling isolated/rejected by husband*.*” *		
